# A Cross-Sectional Study Comparing the Prevalence of Bullous Pemphigoid Autoantibodies in 275 Cases of Type II Diabetes Mellitus Treated With or Without Dipeptidyl Peptidase-IV Inhibitors

**DOI:** 10.3389/fimmu.2019.01439

**Published:** 2019-06-26

**Authors:** Kentaro Izumi, Wataru Nishie, Mutsuo Beniko, Hiroshi Shimizu

**Affiliations:** ^1^Department of Dermatology, Hokkaido University Graduate School of Medicine, Sapporo, Japan; ^2^Department of Diabetes and Endocrinology, Hokkaido P.W.F.A.C. Sapporo Kosei General Hospital, Sapporo, Japan

**Keywords:** bullous pemphigoid, dipeptidyl peptidase-IV inhibitor, autoantibody, ELISA, diabetes mellitus

## Abstract

**Background:** Anti-hyperglycemic drug dipeptidyl peptidase-IV inhibitors (DPP-4i) have recently been recognized as bullous pemphigoid (BP) inducing drugs. It remains uncertain whether DPP-4i induce BP-IgG autoantibodies before the onset of BP.

**Objective:** To evaluate the effect of DPP-4i in the development of BP-IgG autoantibodies in type 2 diabetes mellitus (T2DM) patients.

**Methods:** A cross-sectional study on 221 DPP-4i (+) and 54 DPP-4i (–) T2DM cases was conducted. BP180 NC16A, BP230, and full-length BP180 ELISAs were used to detect the BP-IgG autoantibodies. We have also statistically analyzed the proportion of age, gender, intake periods of DPP-4i, and hemoglobin A1c level between anti-full-length BP180 IgG-positive and -negative DPP-4i (+) T2DM cases to identify co-founding factors.

**Results:** BP180 NC16A ELISA, BP230 ELISA, and full-length BP180 ELISA were positive in 1.8, 2.2, and 10.9% of DPP-4i (+) T2DM cases, respectively; in contrast, they were positive in 0, 7.4, and 5.6% of DPP-4i (–) T2DM cases, respectively. The odds ratio for the development of BP-IgG autoantibodies detected by full-length BP180 ELISA was 2.070 for DPP-4i (+). There were no significant differences between the genders, intake periods of DPP-4i, nor of hemoglobin A1c levels, the anti-full-length BP180 IgG-positive cases tended to be significantly older than anti-full-length BP180 IgG-negative cases (median 74 vs. 69, *p* = 0.025) in the DPP-4i (+) T2DM cases.

**Limitations:** We focused the analysis on DPP-4i intake and not on the effects of metformin and other drugs.

**Conclusion:** Exposure to specific DPP-4i may induce the development of anti-full-length BP180 autoantibodies even in T2DM patients without any clinical symptoms of BP. Aging would be a risk factor to develop anti-full-length BP180-IgG autoantibody in DPP-4i (+) T2DM cases.

## Introduction

Bullous pemphigoid (BP), one of the most common autoimmune blistering skin disorders, is clinically characterized by itchy urticarial erythema and multiple tense blisters (1, 2). BP autoantibodies target two major hemidesmosomal components, BP180 (also known as type XVII collagen or BPAG2) and BP230 (3, 4), and the non-collagenous 16A (NC16A) domain of BP180 is a major pathogenic epitope for BP-IgG autoantibodies (5). Commercial ELISA systems using recombinant BP180 NC16A or BP230 allow BP-IgG to be identified and quantified (6). Although it remains unclear why immunotolerance to these molecules is broken in BP, several triggering factors, including certain drugs (in “drug-induced BP”), have been reported, and more than 50 kinds of drugs have been reported as causative of BP (7). Recently, cases of BP that developed in patients receiving dipeptidyl peptidase-IV inhibitors (DPP-4i) have been increasing (8–16), and a large case-control study from a French pharmacovigilance database also suggested a strong relationship between BP and DPP-4i, especially vildagliptin (17).

DPP-4i are a new class of anti-diabetes drugs that prevent the degradation of glucose-dependent insulinotropic polypeptide and glucagon-like peptide 1 by inhibiting dipeptidyl peptidase-IV (DPP-4) (18). DPP-4i has been regarded as a safe, anti-hyperglycemic drugs because of the low risk of hypoglycemia; therefore, DPP-4i is widely used to treat the type 2 diabetes mellitus (T2DM).

Although it is likely that DPP-4i use carries an increased risk for the development of BP, there is no information about the prevalence of BP among the DPP-4i-receiving T2DM patients. In addition, it is also uncertain whether the use of DPP-4i may induce BP-IgG autoantibodies in T2DM patients without any blister formation as a pre-clinical stage of BP. Here we investigated the prevalence rates and titration of BP-IgG autoantibodies using three different ELISA systems—BP180 NC16A ELISA, full-length BP180 ELISA (BP180-FL ELISA), and BP230 ELISA—for diagnosing BP in 275 cases of T2DM treated with or without DPP-4i.

## Materials and Methods

### Serum Samples

The investigations were conducted as a cross-sectional study, comparing T2DM cases treated with DPP-4i (*n* = 221) to T2DM cases treated without DPP-4i (*n* = 54), from February 9th to November 14th in 2017. All T2DM patients were diagnosed at the Department of Diabetes and Endocrinology, Hokkaido P.W.F.A.C. Sapporo Kosei General Hospital. The hemoglobin A1c (HbA1c) level was measured during T2DM treatment in both the DPP-4i (–) and the DPP-4i (+) T2DM cases. All study procedures using human materials were performed in accordance to the Declaration of Helsinki Principles. This study was approved by the Ethical Committee of Hokkaido University (016-0061), and full informed consent was obtained from all patients and healthy volunteers for the use of their materials.

### Data Collection for Cohorts

The study was conducted at the Department of Dermatology, Hokkaido University Graduate School of Medicine. By using the database of clinical records in the Department of Diabetes and Endocrinology, Hokkaido P.W.F.A.C. Sapporo Kosei General Hospital, we collected basic patient data, past medical histories, and laboratory data.

### Detection of BP-IgG Autoantibodies

We performed conventional BP180NC16A and BP230 ELISAs (MBL, Nagoya, Japan) following the manufacturer's instructions, and we performed BP180-FL ELISA as previously reported to detect BP-IgG autoantibodies in our patient groups (19). Indirect immunofluorescence using 1 M NaCl-split skin was performed on sera that were positive in the above-mentioned ELISAs as previously described (20).

### Statistics

An Unpaired *t*-test and a Mann-Whitney test were used to assess the differences of age, DPP-4i intake period, and level of HbA1c between the DPP-4i (–) and the DPP-4i (+) T2DM cases. Fisher's exact test was used to assess the differences of ELISA positive rates between the DPP-4i (–) and the DPP-4i (+) T2DM cases with GraphPad Prism 7.03. Fisher's exact test was also used to assess the difference of male/female ratio between the DPP-4i (–) and the DPP-4i (+) T2DM cases with GraphPad Prism 7.03. Furthermore, univariate logistic regression was used to analyze associations between reactivity to each BP-IgG and DPP-4i, and to calculate the odds ratios with EZR (Saitama Medical Center, Jichi Medical University, Saitama, Japan), which is a graphical user interface for R (The R Foundation for Statistical Computing, Vienna, Austria) (21). More precisely, it is a modified version of R commander designed to add statistical functions frequently used in biostatistics.

## Results

### Gender, Age, Diabetes Severity, and Treatment Modalities in T2DM Cases With DPP-4i

From February 9th, 2017, to November 14th, 2017, we enrolled 275 T2DM cases without any cutaneous symptoms in this study. Of these, 221 cases were treated with DPP-4i [designated as DPP-4i (+)] whereas the remaining 54 cases were treated without DPP-4i [designated as DPP-4 (–)]. In the DPP-4i (+) group, 38.9% of cases were female, the mean age was 68.0 ± 10.6 years and the mean HbA1c was 7.1 ± 0.9. In the DPP-4i (–) group, 33.3% of cases were female, and the mean age was 68.9 ± 9.0 years. Regarding therapeutic modalities for T2DM in subjects of this study, 46.3% of the DPP-4i (–) group were treated with a diet alone. Sulfonylurea was the most commonly prescribed agent (25.9%), followed by biguanide (24.1%), sodium-glucose transport protein 2 (SGLT2) inhibitor (9.3%), α-glucosidase inhibitor (5.6%), insulin (5.6%), and other (1.9%) in the DPP-4i (–) group, whereas in the DPP-4i (+) group, biguanide was the most commonly prescribed (52.9%) in addition to DPP-4i, followed by sulfonylurea (36.7%), insulin (15.8%), α-glucosidase inhibitor (11.3%), glinide (7.2%), SGLT2 inhibitor (6.8%), thiazolidine (5.4%), and other (6.8%). The mean HbA1c for the DPP-4i (–) group was 6.6 ± 0.5, which was significantly lower than that of the DPP-4i (+) group.

Regarding the medication in the DPP-4i (+) cohort, sitagliptin was the most commonly used (*n* = 87, 39.4%), followed by anagliptin (*n* = 40, 18.1%), vildagliptin (*n* = 37, 16.7%), teneligliptin (*n* = 26, 11.8%), linagliptin (*n* = 21, 10.5%), alogliptin (*n* = 8, 3.6%), saxagliptin (*n* = 1, 0.5%), and omaligliptin (*n* = 1, 0.5%). The mean period of DPP-4i administration was 36.5 ± 24.3 months. There were no significant differences in age or gender between the DPP-4i (+) and the DPP-4i (–) groups; however, HbA1c of the DPP-4i (+) group was significantly higher than that of the DPP-4i (–) group (Table 1).

**Table 1:** Positive rates of BP180 NC16A, BP230, and BP180-FL ELISAs for each DPP-4i drug.

	**BP180 NC16A**	**Odds ratio *p*-value**	**BP230**	**Odds ratio *p*-value**	**BP180-FL**	**Odds ratio *p*-value**
DPP-4i (–) (*n* = 54)	0 (0.0%)	1.000 1.000	4 (7.4%)	1.000 1.000	3 (5.6%)	1.000 1.000
DPP-4i (+) (*n* = 221)	4 (1.8%)	1.580 x 10^−7^ 0.995	5 (2.2%)	0.289 0.071	24 (10.9%)	2.070 0.249
Sitagliptin (*n* = 87)	0 (0.0%)	1.000 1.000	3 (3.4%)	0.446 0.304	11 (12.6%)	2.460 0.183
Anagliptin (*n* = 40)	0 (0.0%)	1.000 1.000	0 (0.0%)	1.460 × 10^−8^ 0.995	2 (5.0%)	0.895 0.906
Vildagliptin (*n* = 37)	2 (5.4%)	3.610 x 10^−8^ 0.998	2 (5.4%)	0.714 0.707	5 (13.5%)	2.660 0.201
Teneligliptin (*n* = 26)	2 (7.7%)	5.260 x 10^−8^ 0.998	0 (0.0%)	1.460 × 10^−8^ 0.996	3 (11.5%)	2.220 0.351
Linagliptin (*n* = 21)	0 (0.0%)	1.000 1.000	0 (0.0%)	1.460 × 10^−8^ 0.996	3 (14.2%)	2.830 0.227
Alogliptin (*n* = 8)	0 (0.0%)	1.000 1.000	0 (0.0%)	1.460 × 10^−8^ 0.998	0 (0.0%)	4.000 × 10^−7^ 0.992
Saxagliptin (*n* = 1)	0 (0.0%)	1.000 1.000	0 (0.0%)	1.460 × 10^−8^ 0.999	0 (0.0%)	4.000 × 10^−7^ 0.997
Omaligliptin (*n* = 1)	0 (0.0%)	1.000 1.000	0 (0.0%)	1.460 × 10^−8^ 0.999	0 (0.0%)	4.000 × 10^−7^ 0.997

### Anti-Full-Length BP180 Autoantibodies Were Highly Detected in the DPP-4i (+) T2DM Cases

**Figure 1:**
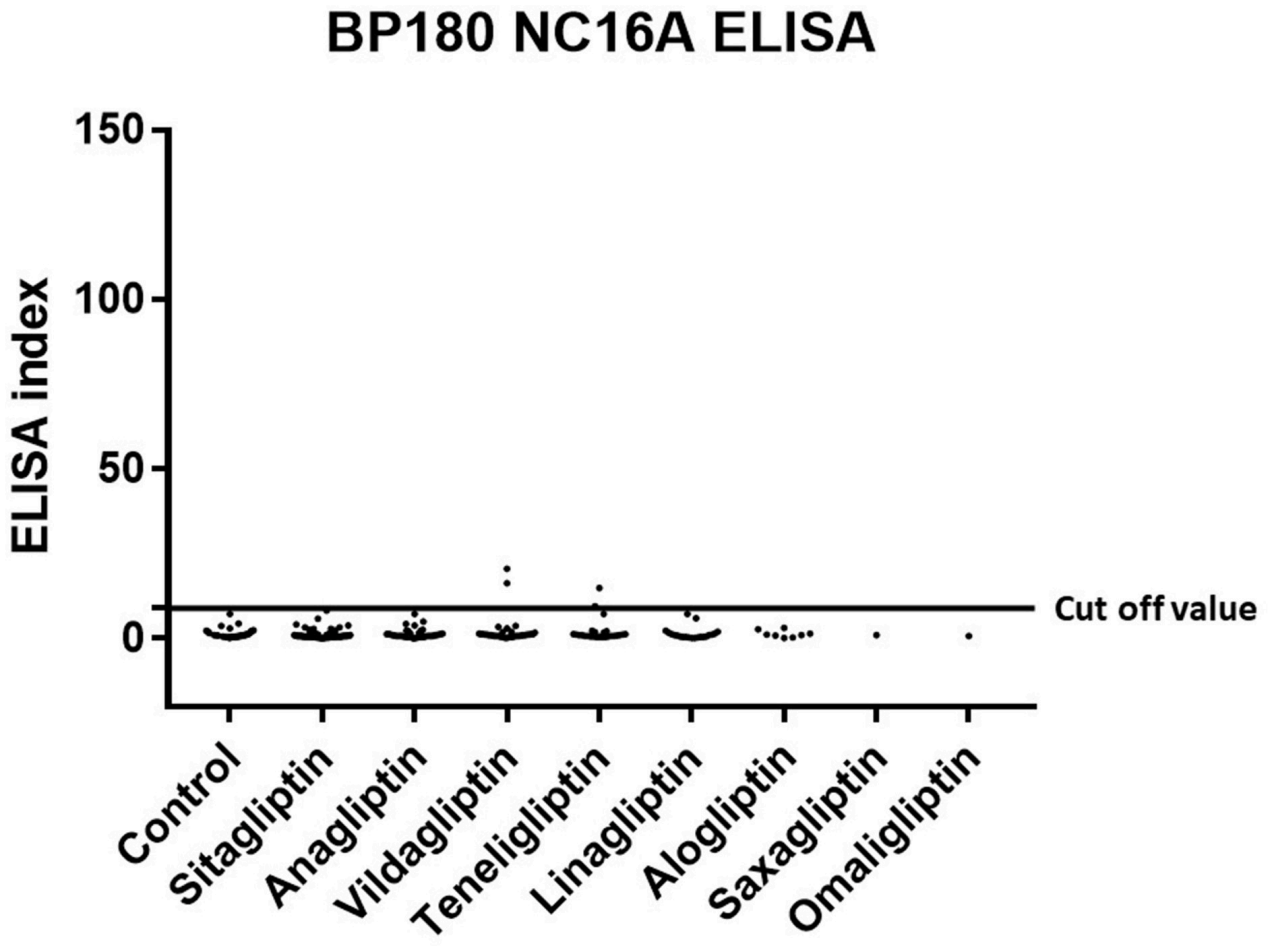
Type 2 diabetes mellitus. Scatter plot of BP180 NC16A ELISA index in DPP-4i (–) and DPP-4i (+). Cut-off value of BP180 NC16A ELISA<9.0, Control indicates the DPP-4i (–) group.

**Figure 2:**
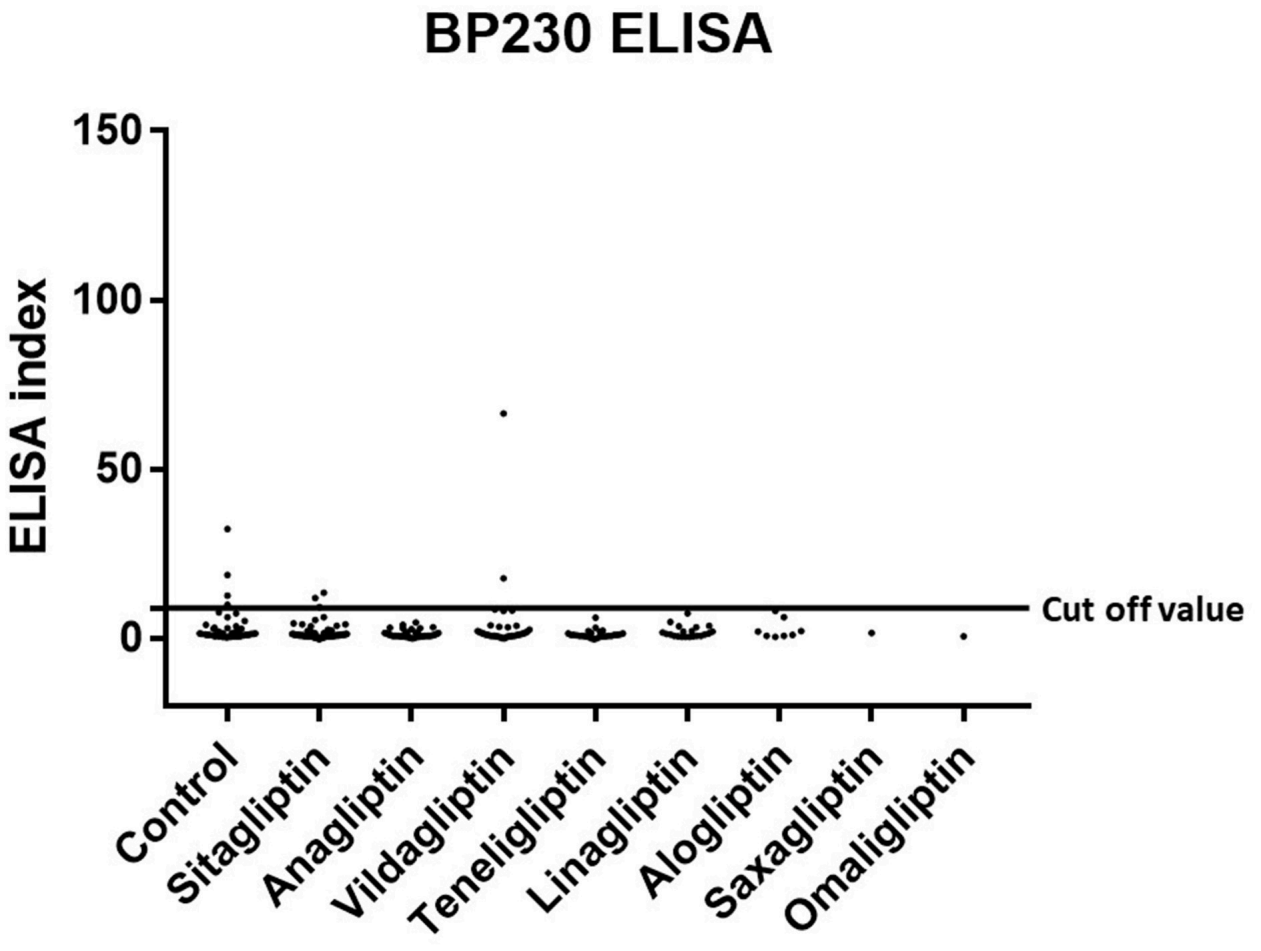
Type 2 diabetes mellitus. Scatter plot of BP230 ELISA index in DPP-4 (–) and DPP-4i (+). Cut-off value of BP230 ELISA<9.0, Control indicates the DPP-4i (–) group.

**Figure 3:**
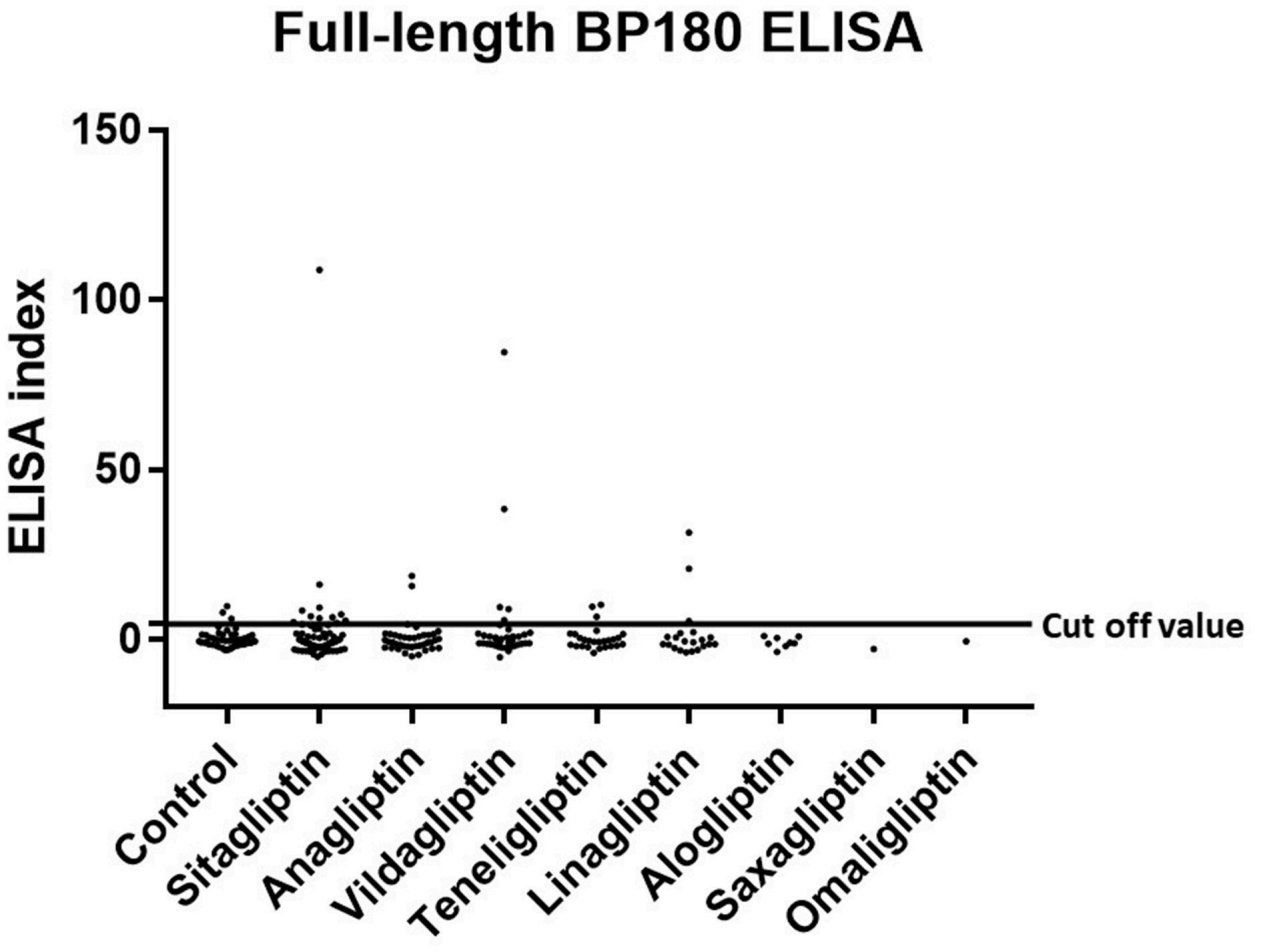
Type 2 diabetes mellitus. Scatter plot of BP180-FL ELISA index in DPP-4i (–) and DPP-4i (+). Cut-off value of BP180-FL ELISA<4.64, Control indicates the DPP-4i (–) group.

Prevalence and titration of BP-IgG detected with BP180 NC16A, BP230, and BP180-FL ELISAs are shown in Table 1 and Figures 1–3. The false-positive rates of BP180 NC16A, BP230, and BP180-FL ELISAs are 1.1, 1.0, and 5.7%, respectively (based on the manufacturer's instructions and our previous report) (19). Therefore, the positive rates of BP-IgG detected with all ELISAs were roughly twice the false-positive rates. Focusing on the BP180-FL ELISA, the ELISA indices of all three anti-full-length BP180 IgG-positive cases in the DPP-4i (–) group were <10.0 (close to the normal range), whereas those of nine out of 24 anti-full-length BP180 antibody-positive cases were higher than 10.0. Indirect immunofluorescence using 1 M NaCl-split human skin for the anti-full-length BP180 revealed that 13 of the 24 sera (54.2%) that were positive in the BP180-FL ELISA had BP-IgG autoantibodies directing the epidermal side of the artificial blisters (not shown). None of the cases showed reactivity against the dermal side. The total and drug-specific prevalence's of each BP-IgG are shown in Table 1. The prevalence of anti-full-length BP180 IgG but not of BP180 NC16A nor of BP230 shows a higher odds ratio for DPP-4i (+) than for DPP-4i (–).

### Aging Was Correlated With the Prevalence of Anti-Full-Length BP180 Autoantibodies in DPP-4i (+) T2DM

To explore the confounding factor between DPP-4i medication and BP-IgG autoantibody development, we compared age, male/female ratio, DPP-4i intake period, and HbA1c (Table 2) in both anti-full-length BP180 IgG autoantibody-positive and -negative cases in the DPP-4i (+) group. Although there were no significant differences between the genders, intake periods of DPP-4i nor HbA1c levels, the anti-full-length BP180 IgG-positive cases tended to be significantly older than anti-full-length BP180 IgG-negative cases (median 74 vs. 69, *p* = 0.025).

**Table 2:** Comparison of age, male/female ratio, DPP-4i intake period, and HbA1c for anti-full-length BP180 IgG autoantibody-positive and -negative cases in the DPP-4i (+) group.

**BP180-FL IgG**	**Negative**	**Positive**	***p*-value**
Age (median)	69^a^	74	0.025^*^
Male/Female ratio	1.63:1	1.18:1	0.509
DPP-4i intake (mean months)	35.6 ± 24.5^b^	43.4 ± 21.2	0.115
HbA1c (mean)	7.1 ± 0.9	7.1 ± 0.8	0.936

a *Age data of one case was not obtained in anti-full-length BP180 IgG-negative cases*.

* *p < 0.05 by Mann-Whitney test*.

b *DPP-4i intake period data of four cases were not obtained for the anti-full-length BP180 IgG autoantibody-negative cases*.

## Discussion

The present study revealed that around 10% of DPP-4i (+) T2DM patients already have BP-IgG autoantibodies directing BP180. The prevalence rate of BP-IgG autoantibodies was much higher than we expected in DPP-4i (+) T2DM patients compared to that of DPP-4i (–). It should also be noted that DPP-4i (+) showed increased odds ratio 2.070 for the development of anti-full-length BP180 IgG autoantibodies compared with that of DPP-4i (–) T2DM patients. The tendency for the prevalence rate of BP-IgG autoantibodies to be high for vildagliptin was similar to the reporting odds ratios as causative drugs analyzed by a large case-control study based on the French Pharmacovigilance Database (17). Notably, five (2.3%) out of the 221 members of the DPP-4i (+) group showed high titrations of anti-full-length BP180 IgG autoantibodies (ELISA index = 108.8, 84.7, 38.4, 31.4, 20.9, respectively), whereas members of the DPP-4i (–) group showed very low titrations close to the cut-off value (ELISA index = 6.0, 8.0, and 9.8) even with a positive reaction. Furthermore, around 50% of cases whose sera showed a positive reaction for the BP180-FL ELISA reacted with the epidermal side of the artificial blisters with indirect immunofluorescence using 1 M NaCl-split human skin. These data suggest that there are quite large numbers of T2DM patients with autoantibodies directing BP180 (19). Although we also evaluated the skin symptoms of the subjects in this research, none of the DPP-4i (+) T2DM cases with BP-IgG autoantibodies showed cutaneous symptoms suggestive of BP. However, the prospective observation of these subjects in the current study could shed insight on the exact rates of prevalence for BP onset and BP-IgG autoantibody profiles in DPP-4i (+) T2DM.

To detect BP-IgG autoantibodies in DPP-4i (+) T2DM cases, we used three different ELISAs (full-length BP180, BP180 NC16A, and BP230) in the present study. As described above, BP180-FL ELISA showed the highest positive rates for detecting BP-IgG autoantibodies in DPP-4i (+) of the three ELISAs. In addition, the positive rates for the BP180 NC16A and BP230 ELISAs were 1.8 and 2.8%, respectively; these percentages were similar to the false-positive rates. These facts suggest that the BP180-FL ELISA might contribute to the detection of BP-IgG autoantibodies in DPP-4i (+) T2DM cases. This fact is also consistent with a previous study which suggested that in BP cases associated with DPP-4i (+) T2DM tends to develop non-NC16A-IgG autoantibodies more frequently than spontaneous BP (19). Unexpectedly, lower positive rates of anti-BP230 IgG were seen in DPP-4i (+) T2DM patients compared with those of DPP-4i (–) T2DM patients. It is well-known that levels of anti-BP180 autoantibodies correlate closely with disease severity in BP, whereas levels of anti-BP230 autoantibodies do not (22). Previous studies have reported on the role of anti-BP180 autoantibodies in blister formation; however, the pathogenicity of anti-BP230 remains controversial (2). Thus, we focused on the prevalence of anti-BP180 IgG, however the increased prevalence rate of anti-BP230 in the DPP-4i (–) group is of interest. Therefore, the prevalence of anti-BP230 IgG in T2DM cases should be addressed in future research.

It is well known that most of the BP sera reacts with the NC16A domain of BP180, and BP-IgG directing NC16A has pathogenicity in BP, while the pathogenicity of anti-non-NC16A BP-IgG is still controversial. To characterize anti-BP180-FL IgG in DPP-4i (+) cases, we performed indirect immunofluorescence using 1 M NaCl-split human skin. As a result, 13 of the 24 sera (54.2%) that were positive in the BP180-FL ELISA had BP-IgG autoantibodies directing the epidermal side of the artificial blisters. This result is in contrast to a previous report that found that none of the BP180-FL ELISA positive patients with Alzheimer's disease without BP symptoms had positive IIF (23). This discrepancy of BP-IgG's binding capacity to the dermo-epidermal junction among anti-non-NC16A-IgG might be induced by the differences of their epitopes. Our previous report showed that around 10% of BP cases, of which sera showed a positive reactivity against only BP180-FL but not BP180-NC16A (19). Furthermore, 50% of this non-NC16A BP were treated with DPP-4i before BP onset. This report suggests that anti-non-NC16A-IgG could also show pathogenicity in BP as well as a potential association between non-NC16A BP autoantibody production and DPP-4i.

Regarding aging, BP is well known to preferentially occur in the elderly. A recent case-control study reported that the effects of DPP-4i on BP onset were significantly associated with the age of 80 years or older (24). These reports are consistent with current data because our data clearly show a correlation between aging and BP-IgG autoantibody development in DPP-4i (+) T2DM cases. Therefore, clinicians should pay particular attention to the development of BP in elderly DPP-4i (+) T2DM cases.

Although evidence has been increasing that DPP-4i is associated with the onset of pemphigoid diseases, including BP and mucous membrane pemphigoid (25), it remains uncertain why DPP-4i causes immune tolerance failure against BP antigens, resulting in BP onset. DPP-4 is ubiquitously expressed on many cells, including keratinocytes, T-cells, and endothelial cells (26, 27). DPP-4 cleaves and inactivates many proinflammatory cytokines as its substrates. Therefore, DPP-4i could promote the activity of proinflammatory chemokines (e.g., eotaxin) and enhance eosinophil activation, resulting in tissue damage and blister formation in the lesioned skin (28). This chemo-attractive function of DPP-4i might contribute to the exacerbation of BP in T2DM cases. Regarding the genetic background of DPP-4i BP, our group reported that HLA-DQB1^*^03:01 is strongly associated with non-inflammatory DPP-4i BP (29). Based on these concepts, DPP-4i could be a causative factor as a trigger of BP onset as well as an exaggerating factor of BP disease activity.

Regarding the prognosis of the BP-IgG-positive cases in DPP-4i (+), the discontinuation of DPP-4i might be recommended to prevent the potential development of DPP-4i-related BP. However, further studies are necessary to establish enough evidence for stopping DPP-4i medication in BP-IgG-positive T2DM cases treated with DPP-4i. We have followed 220 DPP-4i (+) cases for a mean 25.5 ± 0.6 months from blood-taking to the present; however, none of the cases developed BP symptoms. This reminds us that other factors (e.g., epitope spreading), in addition to the presence of BP-IgG, are necessary for BP symptoms to develop.

Our study had some limitations, in that we focused on the analysis of DPP4i intake, while the potential effect of metformin was not investigated. We did not follow the fluctuations of BP-IgG titrations in the enrolled cases. Because of the limited DPP-4i (–) sample number, the statistical power may not be enough to address the prevalence of BP-IgG autoantibodies between the DPP-4i (+) T2DM cases and the DPP-4i (–) group. In addition, a limitation of this research is the small number of DPP-4i (–) cases, because DPP-4i is more commonly used in Japan than America and Europe. Another limitation is that HbA1c and multiple drug usage was statistically higher in the DPP-4i (+) than in the DPP-4i (–), which could be co-founding factors.

In conclusion, our findings in a cross-sectional study confirm the prevalence and titration of the BP-IgG autoantibody in DPP-4i (+) T2DM cases. This study suggests that DPP-4i may induce the production of BP-IgG and increase its titration, especially in anti-full-length BP180, even in the BP pre-clinical stage.

## Data Availability

All datasets generated for this study are included in the manuscript.

## Ethics Statement

All study procedures using human materials were performed according to the Declaration of Helsinki Principles. This study was approved by the Ethical Committee of Hokkaido University (016-0061), and fully informed consent was obtained from all patients and healthy volunteers for the use of their materials.

## Author Contributions

KI, WN, and MB participated in designing the study, generating and gathering the data, and analyzing the data. KI, WN, MB, and HS participated in writing the paper. All authors have approved final version of this paper.

## Conflict of Interest Statement

The authors declare that the research was conducted in the absence of any commercial or financial relationships that could be construed as a potential conflict of interest.

## Abbreviations

BP: bullous pemphigoid
NC16A: non-collagenous 16A
DPP-4i: dipeptidyl peptidase-IV inhibitors
T2DM: type 2 diabetes mellitus
DPP-4: dipeptidyl peptidase-IV
BP180-FL ELISA: full-length BP180 ELISA
hemoglobin A1c: HbA1c
SGLT2: sodium-glucose transport protein 2.
